# Evolution of the vertebrate neurocranium: problems of the premandibular domain and the origin of the trabecula

**DOI:** 10.1186/s40851-017-0083-6

**Published:** 2018-01-09

**Authors:** Shigeru Kuratani, Per. E. Ahlberg

**Affiliations:** 1 Laboratory for Evolutionary Morphology, RIKEN, 2-2-3 Minatojima-minami Chuo-ku, Kobe, Kobe, 650-0047 Japan; 2 Department of Organismal Biology, Subdepartment of Evolution and Development, Uppsala University, Norbyvägen 18A, 752 36 Uppsala, Sweden

**Keywords:** Head mesoderm, Cranium, Cyclostomes, Neural crest, Evolution, Trabecula

## Abstract

The subdivision of the gnathostome neurocranium into an anterior neural crest-derived moiety and a posterior mesodermal moiety has attracted the interest of researchers for nearly two centuries. We present a synthetic scenario for the evolution of this structure, uniting developmental data from living cyclostomes and gnathostomes with morphological data from fossil stem gnathostomes in a common phylogenetic framework. Ancestrally, vertebrates had an anteroposteriorly short forebrain, and the neurocranium was essentially mesodermal; skeletal structures derived from premandibular ectomesenchyme were mostly anterior to the brain and formed part of the visceral arch skeleton. The evolution of a one-piece neurocranial ‘head shield’ in jawless stem gnathostomes, such as galeaspids and osteostracans, caused this mesenchyme to become incorporated into the neurocranium, but its position relative to the brain and nasohypophyseal duct remained unchanged. Basically similar distribution of the premandibular ectomesenchyme is inferred, even in placoderms, the earliest jawed vertebrates, in which the separation of hypophyseal and nasal placodes obliterated the nasohypophyseal duct, leading to redeployment of this ectomesenchyme between the separate placodes and permitting differentiation of the crown gnathostome trabecula that floored the forebrain. Initially this region was very short, and the bulk of the premandibular cranial part projected anteroventral to the nasal capsule, as in jawless stem gnathostomes. Due to the lengthening of the forebrain, the anteriorly projecting ‘upper lip’ was lost, resulting in the modern gnathostome neurocranium with a long forebrain cavity floored by the trabeculae.

## Introduction

**Fig. 1:**
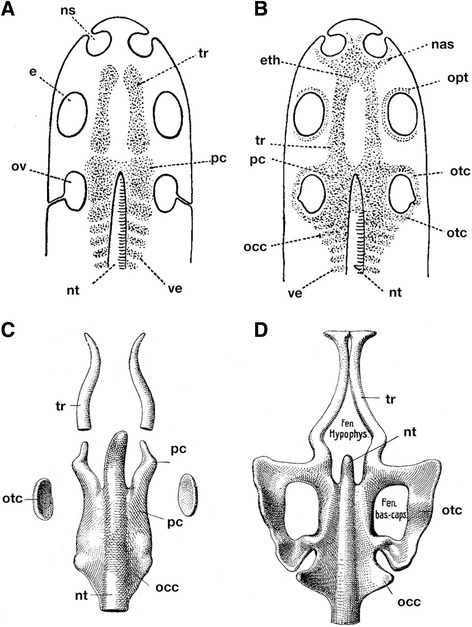
**a** and **b** Schematized composition of crown gnathostome cranium. Early (**a**) and late (**b**) stages of development. Precartilaginous condensation and cartilages are shown in dots. Based on reference [8]. **c** and **d**. Early (**c**) and late (**b**) stages of chondrocranium in *Salmo*. Based on reference [42]. e, eye; eth, ethmoidal plate; Fen. bas.-caps., basicapsular fenestra; Fe, hypophys., hypophyseal fenestra; nas, nasal capsule; ns, nasal sac; nt, notochord; occ, occipital cartilage; opt, optic capsule; otc, otic capsule; ov, otic vesicle; pc, parachordals; tr, trabecula; ve, vertebrae

Ever since Rathke [1], who found a discontinuity between the rostral and caudal part of the cranium, a part of the gnathostome neurocranium known as the trabecula cranii has drawn the attention of vertebrate morphologists (Fig. 1). The difference between the anterior and posterior portions of the neurocranium resides primarily in their morphological relationships; specifically, whether or not the cranial base is medially associated with the notochord, in a manner similar to the vertebral column. The morphological interpretation of this division is profoundly related to the segmental theory of the vertebrate head, developed from Goethe’s and Oken’s ideas about the skull being composed of serial homologues of vertebrae [2–4]. Gegenbaur and Froriep thought that only the posterior part of the gnathostome neurocranium, which lies alongside the notochord, could be compared with the vertebral column; the neurocranium could thus be divided into prespinal and spinal parts [5, 6].

**Fig. 2:**
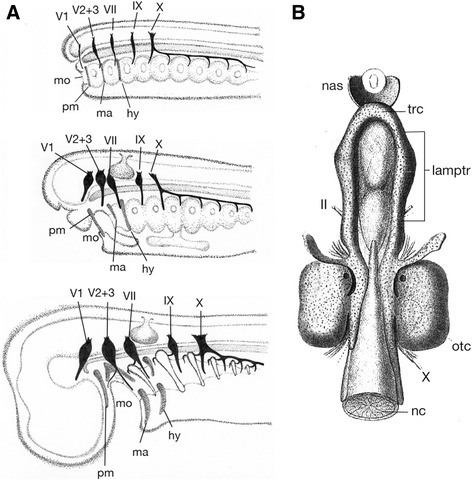
Evolution of the vertebrate neurocranium and origin of trabecula. **a** Scenario by de Beer. From top to bottom, amphioxus-like vertebrate ancestor, ammocoete larval-like intermediate state, and gnathostome morphotype resembling an elasmobranch pharyngula. The trabecula is assumed to represent the premandibular arch, which was secondarily incorporated into the neurocranium of the gnathostome whose forebrain is expanded enormously. Based on reference [11]. **b** The neurocranium of the adult lamprey, which is primarily formed of an inverted U-shaped ‘lamprey trabecula’, rostrally connected with its counterpart by means of the rostralmost portion called, in the present paper, the ‘transverse commissure’. The latter part is assumed to be of premandibular mesoderm and homologous with the orbital (acrochordal) cartilage of jawed vertebrates, and the rest of lamprey trabecula is thought to correspond to a rostrally extended parachordal. This figure is based on reference [31]. Abbreviations: hy, hyoid arch; II, optic nerve; IX, glossopharyngeal nerve; lamptr, lamprey trabecula; ma, mandibular arch; mo, mouth; nas, nasal capsule; nc, notochord; otc, otic capsule; pm, hypothetical premandibular arch by De Beer [11]; trc, transverse commissure; V1, ophthalmic nerve; V2 + 3, maxillomandibular nerve; VII, facial nerve; X, vagus nerve

As recognized by Rathke, the above noted two regions initially arise as two pairs of rod-like cartilages, trabeculae and parachordals (Fig. 1). It was Thomas Huxley who first put forth the idea that the trabecula might represent a highly modified element originally belonging to a visceral arch situated rostral to the mandibular arch (premandibular arch) [7]. In the early twentieth century, the original idea of Huxley was elaborated by many morphologists, including Goodrich and Sewertzoff [8–10]. De Beer also illustrated a schematic diagram to explain how an ancestral amphioxus-like animal evolved into the gnathostome morphotype (Fig. 2a) [11]. This ancestor is assumed to have possessed a series of visceral arches along the anteroposterior axis, and these arches showed no clear differentiation. Of these, the rostral three arches represent prospective premandibular, mandibular and hyoid arches. On the other hand, the shark pharyngula-like gnathostome embryo has the trabecula, functioning as a floor for the expanded forebrain, derived from the premandibular arch. The ammocoete larva-like creature is placed in the middle as an intermediate stage, where the premandibular arch is positioned rostral to the mandibular arch.

**Fig. 3:**
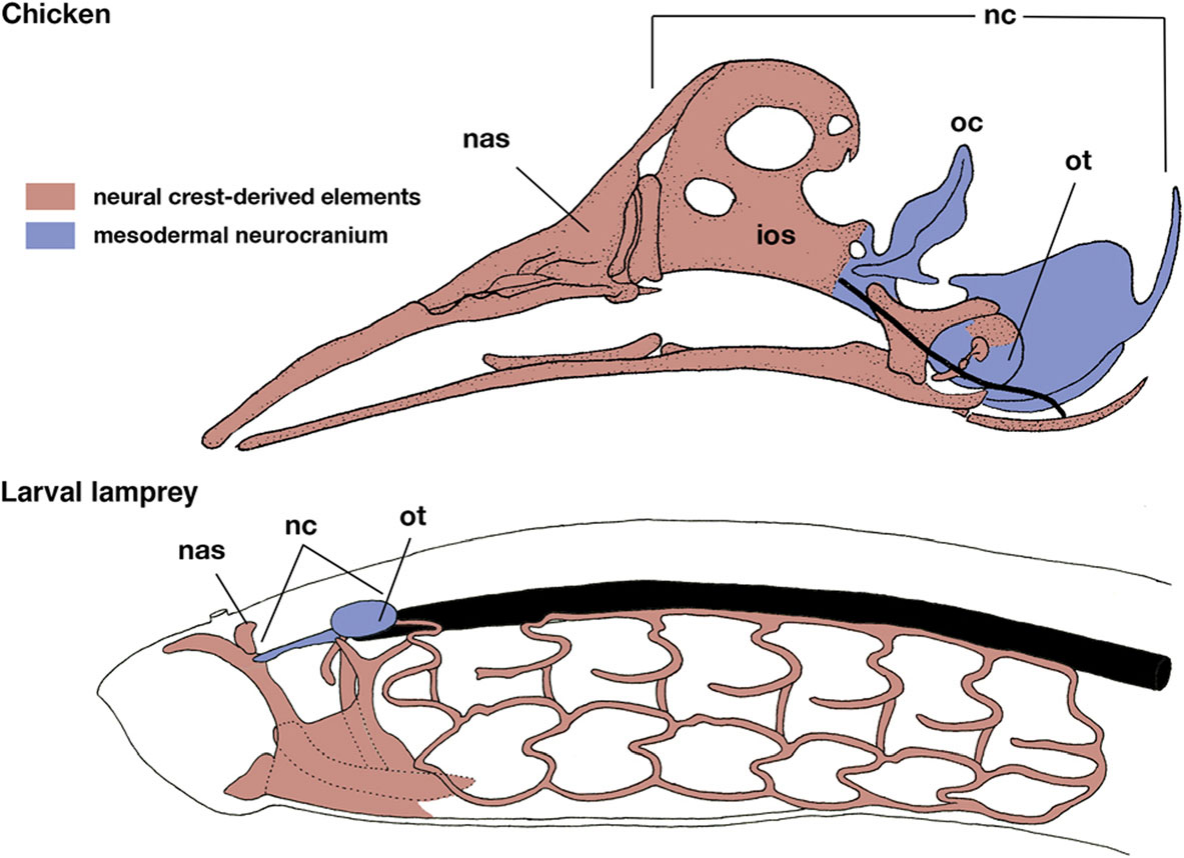
Comparison of ectomesenchyme-mesoderm distribution in the cranium between the crown gnathostome (chicken; based on the description by Couly et al., 1993) and the lamprey larva. Modified from reference [24]. Crest-derived cells are shown in red, mesodermal cells in blue. Notochord is represented by thick black lines. Note that the chordal cranium is coextensive with the notochord. In the chicken, the prechordal cranium is represented by the interorbital septum, whereas in the lamprey, the ectomesenchymal cranium is exclusively oro-pharyngeal, and the neurocranium is made of mesoderm. Ios, interorbita septum; nas, nasal capsule; nc, neurocranium; ot, otic capsule

Toward the end of the 20th century, the concepts developed by Gegenbaur and Froriep gained support from experimental embryology, which showed that the trabecular part of the neurocranium is of cephalic neural crest origin [12, 13], and only the notochord-associated posterior part of the neurocranium is of mesodermal origin, like the vertebral column (Fig. 3). Thus the neural crest/mesoderm distinction does not coincide with the functional distinction of the neurocranium versus viscerocranium. It was further suggested that the paraxial mesoderm requires a signal emanating from the notochord to differentiate into the skeletal tissues, whereas the cephalic crest cells can differentiate without this signal. On this basis, Couly and his colleagues designated the anterior and posterior parts of the neurocranium as the prechordal and chordal cranium, respectively [12]. The two types of mesenchymal cell lineages correspond perfectly to the presence/absence of the notochord, distinct molecular basis of developmental signaling, as well as two types of the neurocranial anlagen. Moreover, the neural crest generally contributes to the visceral part of the cranium (visceral arch skeletons), making the premandibular arch-hypothesis of Huxley [7] appear somewhat plausible, although the basic idea of ‘arches rostral to the mandibular arch’ is currently not supported by many morphologists, mainly due to the apparent lack of pharyngeal pouch-like structures rostral to the mandibular arch.

Although this theoretical framework for the interpretation of the gnathostome neurocranium has a prominent evolutionary dimension, made explicit in De Beer’s schematic diagram (Fig. 2a), it was developed almost entirely without reference to the fossil record. During the late 19th and early twentieth century, fossils of primitive armoured jawed vertebrates (placoderms) and jawless vertebrates (ostracoderms) had already been discovered in rocks from the Devonian period (419–359 million years ago), but they were known only from their external anatomy and were thus essentially uninformative about neurocranial evolution. However, starting with Erik Stensiö’s groundbreaking investigations of ostracoderm and placoderm cranial anatomy by serial grinding and the construction of wax models [14–16], a series of publications have illuminated the neurocranial anatomy of early vertebrates using the techniques of serial grinding, acid preparation, and more recently synchrotron microtomography [17–22].

**Fig. 4:**
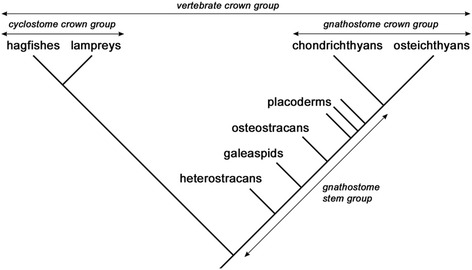
Phylogeny showing the fossil groups discussed in this paper and their relationship to extant gnathostomes and cyclostomes. Double-headed arrows indicate the extent of groups. A stem group contains only fossil taxa; a crown group is defined on the basis of living taxa, but can also include fossils. Heterostracans, galeaspids, osteostracans and placoderms are thus stem gnathostomes but crown vertebrates

These fossils provide detailed data on the shape of the cranial cavity, including the position and approximate morphology of the nasal sacs, inner ears, hypophysis, pineal and parapineal organs, cranial nerves and cranial blood vessels, as well as external features of the neurocranium, such as the articulation points for the visceral arch skeleton. Together these structures create a network of landmarks that can be used for drawing inferences about neurocranial composition, by comparison with developmental data from extant vertebrates. This approach has been greatly strengthened by the discovery that major parts of early cranial development, including the patterns of neural crest cell migration and distribution, are conserved between cyclostomes and gnathostomes [23, 24]. The fossil ostracoderms and placoderms all belong to the gnathostome stem group (Fig. 4), and thus fall within the ‘phylogenetic bracket’ of extant cyclostomes and gnathostomes [25], meaning that the inferences are methodologically robust.

The present review is intended to revisit this developmental morphological issue from an evolutionary standpoint, incorporating fossil data from stem gnathostomes and developmental data from cyclostomes, and to put forth a hypothesis that the ancestral vertebrate cranium may have shown a simpler pattern of development, in which the neurocranium (sensory capsules excluded) was purely mesodermal in origin, and the cephalic neural crest cells contributed only to the oro (naso)-visceral part of the head.

## The cyclostome neurocranium

The heads of cyclostomes (lampreys and hagfishes) differ from those of gnathostomes in many ways other than the obvious absence of jaws. Notably, the forebrain of cyclostomes is anteroposteriorly shorter, the distance between the hypophysis and nasal sacs is smaller, and these organs are located in a common median nasohypophyseal duct [23, 24, 26, 27]. It has already been pointed out that the cephalic crest-derived premandibular ectomesenchyme should be found in the dorsal part of the lamprey oral apparatus, rather than ventral to the forebrain as in a gnathostome [27–29]. However, De Beer [11, 30] was correct in that the lamprey has a less expanded forebrain, and thus a rostral notochordal tip that reaches closer to the rostral end of the head, than a gnathostome. In an ammocoete-like embryo, or a hagfish embryo, it is therefore expected that the neurocranium will be predominantly chordal (of paraxial mesodermal origin) and that the trabecular homolog will not take part in the formation of the neurocranium (see below).

In the lamprey cranium also, traditional comparative morphology has described an inverted U-shaped cartilage called the trabecula (Fig. 2b) [23, 24, 27, 31–34]. It extends rostrally beyond the notochordal tip to the forebrain level and encircles the hypophysis. This structure, however, is not identical to the ‘premandibular arch’ in the ammocoete-like ancestor illustrated by De Beer as in Fig. 2a, nor the premandibular ectomesenchyme that forms the upper lip of ammocoetes. Indeed, it has been shown that the lamprey trabecula represents a rostrally extended parachordal, the paraxial mesodermal cartilage: the earliest anlage of the trabecula is found at the level of the mandibular arch, which grows secondarily rostrally during development [32, 34].

The developmental origin of the transverse commissure is not known; however, it is conceivable that it represents the rostralmost component of the mesodermal neurocranium; the rostralmost paraxial mesoderm in gnathostome embryos is also connected in the midline with its counterpart, just rostral to the notochord reviewed by [35]. The similarity of the morphological configuration between the premandibular mesoderm (cavity) in gnathostomes and transverse commissure in the lamprey supports the above inference, which, however, needs to be tested experimentally. Rostral to the lamprey trabecula is the nasal capsule that is thought to be of neural crest origin (ectomesenchyme in the “anterior process”) [23, 27].

Much less is known about hagfish cranial development [33, 36–38]. However, it has been suggested that, similar to the lamprey, longitudinal rod-like cartilages forming a functional neurocranium are derived from parachordal head mesoderm, and rostrally connect to the oral cartilages derived from the neural crest [23]. The apparent homolog of the ammocoete transverse commissure would be found in a cartilaginous bar, called the hypophyseal commissure, located slightly rostral to the hypophysis, showing a topographical relationship similar to that seen in the lamprey larva. Therefore, the position of this commissure may indicate the rostral limit of the mesodermal neurocranium in the hagfish, as has already been represented in a diagram in a previous paper (Fig. 10 in Reference [23]).

Thus, apart from the crest-derived nasal capsule, the cyclostome neurocranium is mostly formed of mesodermal mesenchyme, and there appears to be no distinct anterior crest-derived neurocranial moiety as seen in the gnathostome chondrocranium (Fig. 3). The question now is, whether the vertebrate neurocranium was composed of crest-derived and mesodermal moieties from the very beginning of their history, or whether the crest-derived part is a gnathostome innovation.

## The fossil evidence

The most detailed fossil evidence about the neurocranium of early vertebrates comes from the jawless osteostracans [14, 16, 18, 39] and galeaspids [20], and the jawed placoderms [15, 17, 21, 22]. These groups all have perichondrally ossified braincases, which preserve the morphology of the cranial cavity, nerve canals and vascular canals. Another fossil jawless group, the heterostracans, lack perichondral ossification, but show faint impressions of the brain, semicircular canals, and branchial pouches on the inner surface of the dermal bones of the head shield [39]. As mentioned above, all these fossil groups belong to the gnathostome stem group. In the older literature the jawless stem gnathostomes are often referred to, collectively, as ‘ostracoderms’ (see above). The uppermost part of the stem is composed of the placoderms, which are themselves paraphyletic (i.e. some placoderms are more closely related to crown gnathostomes than others) [21, 22, 40]. Below them sit the osteostracans, followed by the galeaspids; both groups are monophyletic (Fig. 4). Together, these fossil groups straddle the transition from jawless to jawed vertebrates.

**Fig. 5:**
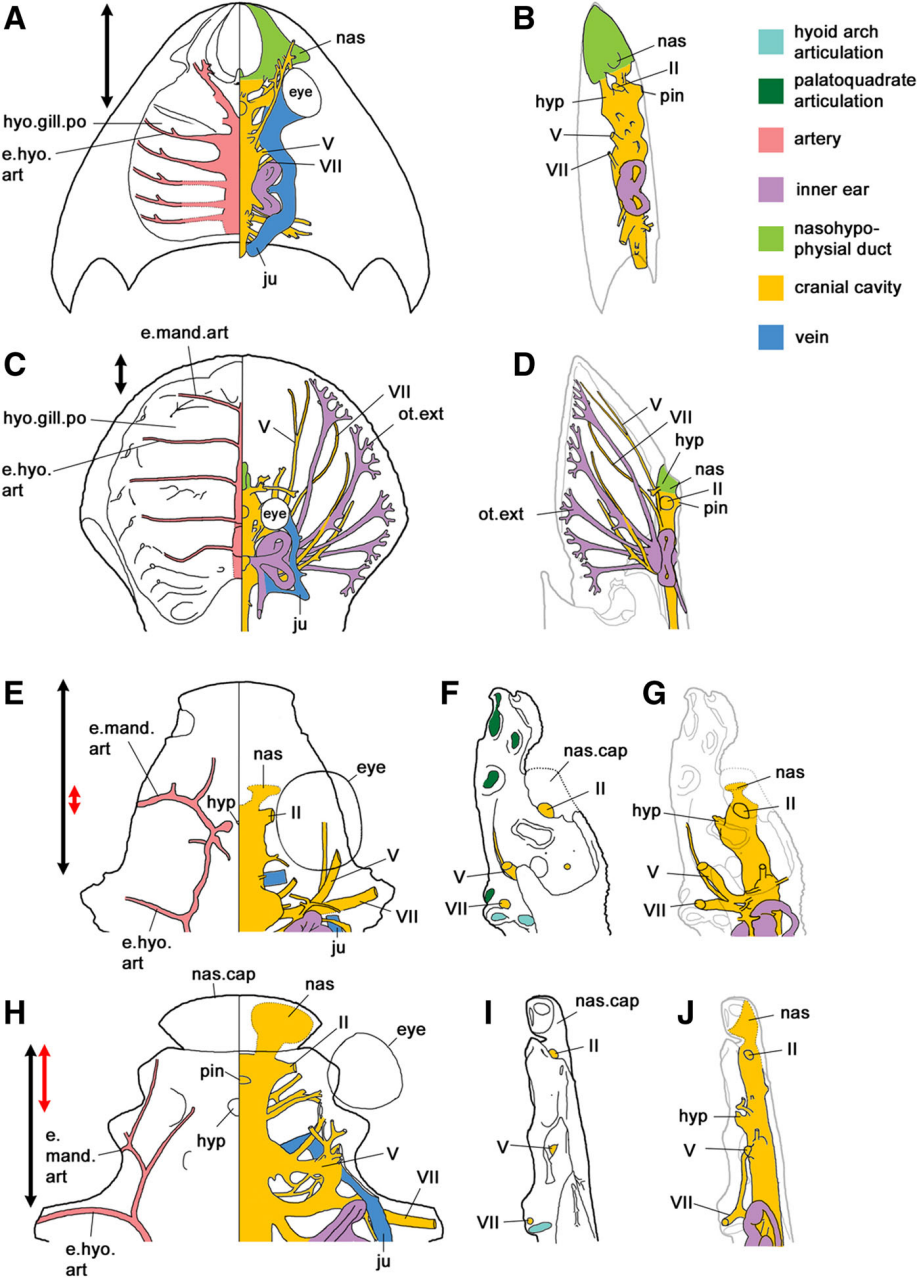
Cranial anatomy of stem gnathostomes. **a**–**b** The galeaspid *Shuyu*, composite reconstruction based on reference [20]. **a** Combined ventral (left) and dorsal (right) image showing the arteries of the pharynx on the left, and the cranial cavity and associated structures on the right. **b** Lateral view showing cranial cavity, nasohypophyseal duct and inner ear. The double-headed black arrow indicates the length of the mandibular-premandibular region, measured from the anterior margin of the spiracular gill pouch to the tip of the snout. **c**–**d** The osteostracan *Norselaspis*, composite reconstruction based on reference [19]. In (**c**), note the anterior position of the pharynx compared to *Shuyu*, and the expansion of the saccular region of the inner ear into multiple long, branched canals (ot.ext) with a presumed sensory function. **e**-**g** The acanthothoracid (primitive placoderm) *Romundina*, redrawn from reference [22]. In this and the next taxon, only the anterior half of the neurocranium is shown. **f** Neurocranium in lateral view. **g** Cranial cavity in lateral view. In (**g**), note that the projecting precerebral part of the neurocranium is ventral to the cranial cavity. The nasal (or rostronasal) capsule is separated from the neurocranium by a fissure. The double-headed red arrow indicates the part of the neurocranium that floors the cranial cavity between the hypophysis and nasal sacs, and can thus be considered a trabecular region sensu stricto. Note that the inferred length of the mandibular-premandibular region (black arrow) is based on the position of palatoquadrate articulations and the exit of the hyomandibular trunk of n VII, and thus relates to the lateral margin of the neurocranium; in the midline the parachordal domain probably extends further forward. **h-j** The arthrodire (derived placoderm) *Kujdanowiaspis*, composite reconstruction based on reference [15]. The precerebral part of the neurocranium has been lost. As in *Romundina*, a fissure separates the rostronasal capsule from the neurocranium proper. e.hyo.art, efferent hyoidean artery; e.mand.art, efferent mandibular artery (generally termed efferent pseudobranchial artery in early gnathostomes); hyo.gill.po, hyoidean gill pouch; hyp, hypophysis; ju, dorsal jugular vein; nas, nasal sac; nas.cap, (rostro) nasal capsule; ot.ext., extension of the inner ear; pin, pineal foramen; II, optic nerve; V, trigeminal nerve; VII, hyomandibular branch of facial nerve

Osteostracans and galeaspids have broad ‘head shields’ that in fact incorporate the entire craniopharyngeal region as far back as the pectoral fins (if present). The neurocranium, which is not subdivided by sutures, is very broad and covers the entire dorsal surface of the gill region. Both groups have a nasohypophyseal duct with a dorsally positioned external opening. The hyoidean branchial pouch appears to have been developed into a normal full-size gill, similar to those on the branchial arches (Fig. 5).

Past analyses of the fossil jawless vertebrates have been greatly influenced by the perception that osteostracans are essentially ‘lampreys in armour’. This idea originated from the observation that the cranial cavity ends anteriorly in a small, ventrally closed nasohypophyseal duct, which opens between the eyes in a lamprey-like manner [14, 16, 18, 39]. However, the positional relationship of the pharynx to the cranial cavity and nasohypophyseal duct is radically different in osteostracans compared to lampreys and other vertebrates (Fig. 5).

The branchial apparatus of osteostracans is displaced so far anteriorly that not only the hyoid gill pouch but also the first two branchial gill pouches lie anterior to the eyes [14, 16, 39]. This uniquely specialized morphology means that the nasohypophyseal duct cannot have a ventral opening onto the palate, because the position that this opening would occupy is already filled by the dorsal aorta of the branchial apparatus (see Fig. 349a in Reference [39]). The apparent similarity with the ventrally closed nasohypophyseal duct of a lamprey, which does not have this positional relationship to the gills, is thus probably convergent.

The specialized pharyngeal morphology of osteostracans limits their utility for investigating the evolution of the cranial cell population map. If we assume that the trigeminal crest cells descended in their customary place, between the optic and otic vesicles, we are forced to conclude that the resulting premandibular and mandibular ectomesenchyme underwent a dramatic forward migration to reach its final destination. The picture is made still more complicated by fan-shaped arrays of canals, emanating from the saccular regions of the inner ears, which terminate near the lateral margins of the head shield and are believed to have had a sensory function (Fig. 5c, d). As the inner ears of living vertebrates are always enclosed within the mesodermal otic capsule, these canal arrays suggest that otic capsule mesoderm extended laterally, above the branchial ectomesenchyme of the gill region, almost to the edges of the shield. However, it is impossible to map the precise boundaries between the premandibular ectomesenchyme (prechordal part of the neurocranium) and mesoderm (chordal cranium), let alone between different components of the ectomesenchyme.

By contrast, galeaspids, heterostracans and placoderms all have hyoid- and branchial arches posterior to the eye. There is some evidence that heterostracans had a hagfish-like nasohypophyseal duct that opened anteriorly [39], but as their neurocranial anatomy cannot be reconstructed in detail they will not be considered further. In galeaspids the nasohypophyseal duct, which is large and either oval or slit-shaped, opens dorsally on top of the head in front of the eyes, and ventrally on the palate (Fig. 5a, b). The nasal sacs and hypophysis open into the duct [20]. Interestingly, the anteriorly directed hypophysis and the olfactory tracts are separated by neurocranial tissue as in crown gnathostomes, which forms a short spike projecting into the nasohypophyseal duct. This spike has been interpreted as a rudimentary trabecula [20]. Its existence suggests that galeaspids had separate nasal and hypophyseal placodes, rather than a single nasohypophyseal placode as in cyclostomes, allowing premandibular ectomesenchyme to differentiate into primitive prechordal cranium that grew forward between the placodes.

Galeaspids, osteostracans and the majority of placoderms all have very short forebrains, with the result that the cranial cavity hardly extends in front of the eyes [14–18, 21, 22, 39]. This resembles the condition in extant cyclostomes and most probably represents retention of the primitive vertebrate condition. Nevertheless, the broad and slab-like neurocrania of galeaspids and osteostracans extend not only laterally to cover the whole branchial region, but also anterior to the eyes and nasohypophyseal duct, right to the tip of the snout (Fig. 5). This preorbital region effectively corresponds to the lamprey upper lip [27]. In galeaspids the entire preorbital region lies anterior to the first pharyngeal pouch, and is presumably composed of some combination of mandibular and premandibular ectomesenchyme; in osteostracans it may also incorporate hyoid and even branchial ectomesenchyme, due to the anterior displacement of the pharynx. In other words, the neurocranium of these jawless stem gnathostomes incorporates an anterior component derived from premandibular ectomesenchyme, but unlike in crown gnathostomes it does not underlie the brain as the trabecula.

Placoderms have separate left and right nasal sacs opening onto the face, and an adenohypophysis opening onto the palate, in the typical gnathostome manner; there is no nasohypophyseal duct. As mentioned above, the forebrain cavity is usually very short. It is floored by a part of the neurocranium that lies in front of the buccohypophyseal foramen, medial to the palatoquadrates, and can thus be identified as a trabecular region (Fig. 5). Compared to crown gnathostomes this region is very broad, reflecting the generally broad and flat character of the neurocranium (probably a primitive character retained from jawless ancestors; see above).

Interestingly, its length is highly variable. In arthrodire placoderms (Fig. 5h-j), and in the so-called ‘maxillate placoderms’ which may be close to the origin of osteichthyans, the nasal capsules are positioned terminally on the snout and the trabecular region is short, reflecting the short forebrain cavity [15, 19, 39, 40]. However, in a few placoderms including the acanthothoracid *Romundina* [21, 22], the trabecular region is developed into a projecting ‘upper lip’ that extends forward below and in front of the nasal capsules (Fig. 5e-g). Its ventral position, and the fact that it is flanked throughout its length by the palatoquadrates, indicates that it is composed of premandibular infraoptic ectomesenchyme, the anlage that gives rise to the upper lip in the lamprey. In these placoderms the crest-derived part of the neurocranium is thus largely, but not entirely, anterior to the cranial cavity. The similarity with jawless vertebrates, especially galeaspids, is obvious and probably indicates that this is a retained primitive character [21].

It seems that the origin of the crown gnathostome trabeculae involved several steps and provides an example of how a seemingly trivial anatomical modification can open the door to major morphological change. In a cyclostome, the forebrain cavity is floored only by the soft dorsal wall of the nasohypophyseal duct. The first step in the origin of the trabeculae, starting perhaps already in galeaspids but brought to completion in early placoderms, was the re-routing of at least part of the premandibular ectomesenchyme into the space between the hypophyseal and nasal placodes. This obliterated the nasohypophyseal duct (although the buccohypophyseal foramen can be considered a remnant of its ventral opening on the palate), and gave the forebrain cavity a skeletal floor [41].

Initially the forebrain remained very short, as did the trabecular region sensu stricto, i.e. the part of the prechordal neurocranium that floors the forebrain cavity. The loss of the projecting premandibular “upper lip” in arthrodires and maxillate placoderms was not immediately accompanied by lengthening of the forebrain, but simply led to a shortening of the preorbital face. However, the fact that the forebrain was now enclosed in a protective skeletal box meant that it could be lengthened without deleterious effects, in a way that the unprotected forebrain of a cyclostome cannot. Within a relatively short time period, an elongated forebrain evolved at least three times in parallel in early jawed vertebrates; in tapinosteid arthrodires, in macropetalichthyid placoderms, and in crown gnathostomes [15, 21].

## Conclusions

**Fig. 6:**
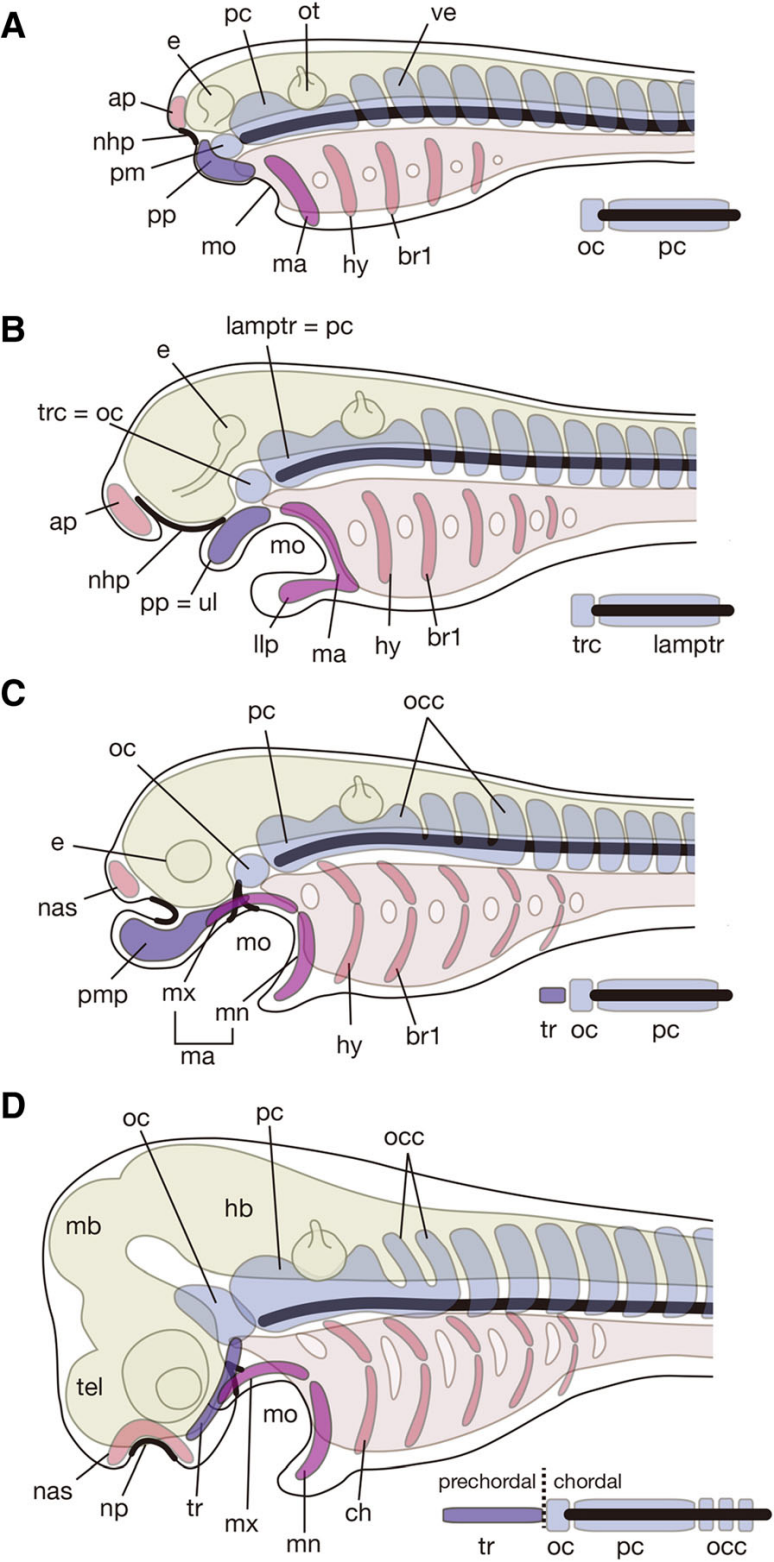
Evolution of the vertebrate neurocranium to explain the establishment of composite neurocranium, as a series of evolutionary grades from a simple primitive ancestor (**a**) to modern gnathostomes (**d**). Cephalic neural crest-derived skeletal elements are colored pink except for the mandibular arch- and posterior process ectomesenchyme colored darker pink and purple, respectively. Paraxial mesodermal elements are colored light blue. For every evolutionary stage, diagrammatic composition of the neurocranium is presented on the lower right. **a** Hypothetical ancestral condition. The forebrain is only weakly developed, and the paraxial mesoderm is coextensive with the notochord that extends almost to the rostral tip of the body axis. Around the neural tube, the neural crest-derived ectomesenchyme is only seen around the nasal-hypophyseal placodes (nhp) as anterior and posterior processes (ap, pp). The neurocranium of this animal (only the part that covers the brain, excluding the nasal capsule) is formed only of the mesodermal tissue, consisting of the premandibular mesoderm-derived element and the parachordal. The neural crest is mostly forming oro-visceral skeleton. The rostral ectomesenchyme in the premandibular domain (pp) may have been utilized to form a part of the oral apparatus. **b** Idealized monorhinous jawless state, corresponding to modern cyclostomes and possibly stem gnathostomes as well. The premandibular ectomesenchyme is utilized as a part of the oral apparatus, like the upper lip of the lamprey larva (ul). **c** Early jawed stage as seen in a basal placoderm like *Romundina* [21]. The mandibular arch is dorsoventrally divided to differentiate into the upper jaw (mx) and the lower jaw (mn). **d** Idealised embryonic scheme of crown gnathostomes based on the elasmobranch morphology. The premandibular ectomesenchyme (corresponding to the posterior process in the ancestor) is now forming a rostral part of the neurocranium to support the expanded forebrain, and the entire neurocranium consists of a prechordal region derived from the neural crest (trabecula) and a chordal region derived from the mesoderm (orbital cartilage and parachordals). The importance of the prechordal cranium increases with the expansion of the forebrain. Note, in (**c**) and (**d**), that the posterior part of the chordal cranium has incorporated an occipital component, derived from a few rostral somites. ap, anterior process; br1, branchial arch 1; e, eye; hb, hindbrain; hy, hyoid arch; lamptr, lamprey trabecula; llp, lower lip; ma, mandibular arch; mb, midbrain; mn, mandibular process; mo, mouth; mx, maxillary process; nas, nasal capsule; nhp, nasohypophyseal plate; oc, orbital cartilage; occ, occipital cartilage; ot, otic vesicle; pc, parachordal cartilage; pm, premandibular mesoderm; pmp, premedian plate; pp., posterior process; tel., telencephaon; tr, trabecula; trc, transverse commissure in the lamprey (hypophyseal commissure in the hagfish); ul, upper lip

Combining the evidence from living and fossil vertebrates, it is possible to construct a scenario for the evolution of the gnathostome neurocranium (Fig. 6). The last common ancestor of gnathostomes and cyclostomes probably had a short forebrain, and the premandibular ectomesenchyme was restricted to the preorbital (and precerebral) part of the head. If this ancestor had a cyclostome-like neurocranium, rather than the shield-shaped type of neurocranium seen in osteostracans and galeaspids, it is likely that its premandibular ectomesenchyme contributed only to the oropharyngeal skeleton and not to the neurocranial base. Thus, the primitive neurocranium was mostly mesodermal in origin. In the gnathostome stem group, the evolution of a shield-shaped neurocranium extending all the way to the tip of the snout led to the incorporation of the premandibular ectomesenchyme into this co-ossified structure, but it was still restricted to the region anterior to the nasohypophyseal duct, as a part of the oral apparatus, and did not floor any part of the cranial cavity. The redeployment of part of this ectomesenchyme to the space between the hypophyseal and nasal placodes, approximately at the same time as the origin of jaws, led to the loss of the nasohypophyseal duct and the creation of a trabecular floor of the forebrain cavity. At first, as exemplified by the placoderm *Romundina*, this trabecular region sensu stricto was only a small posterior part of the premandibular ectomesenchyme territory. Subsequent loss of the projecting ‘upper lip’, together with lengthening of the forebrain, led eventually to the characteristic neurocranium of crown gnathostomes, with a trabecular region of ectomesenchymal origin flooring the prechordal part of the cranial cavity (prechordal cranium). The curious dual nature of the neurocranium, first noted by Rathke nearly two centuries ago, seems to be a byproduct of the transition from jawless to jawed vertebrates.
